# Nonparametric Facial Feature Localization Using Segment-Based Eigenfeatures

**DOI:** 10.1155/2016/6730249

**Published:** 2015-12-24

**Authors:** Hyun-Chul Choi, Dominik Sibbing, Leif Kobbelt

**Affiliations:** ^1^Department of Electronic Engineering, Yeungnam University, 280 Daehak-Ro, Gyeongsan, Gyeongbuk 38541, Republic of Korea; ^2^Computer Graphics and Multimedia Group, RWTH Aachen University, Lehrstuhl für Informatik 8, 52056 Aachen, Germany

## Abstract

We present a nonparametric facial feature localization method using relative directional information between regularly sampled image segments and facial feature points. Instead of using any iterative parameter optimization technique or search algorithm, our method finds the location of facial feature points by using a weighted concentration of the directional vectors originating from the image segments pointing to the expected facial feature positions. Each directional vector is calculated by linear combination of eigendirectional vectors which are obtained by a principal component analysis of training facial segments in feature space of histogram of oriented gradient (HOG). Our method finds facial feature points very fast and accurately, since it utilizes statistical reasoning from all the training data without need to extract local patterns at the estimated positions of facial features, any iterative parameter optimization algorithm, and any search algorithm. In addition, we can reduce the storage size for the trained model by controlling the energy preserving level of HOG pattern space.

## 1. Introduction

The vision-based face monitoring became one of the convenient human-computer-interaction (HCI) tools since face region detection and tracking algorithms [1–3] have been proposed. To realize HCI tool for mobile devices, we still need a low time and memory consuming technique to avoid heavy load in pattern searching or matching algorithm.

In this paper, we adopt the approach of [4] which uses regularly distributed image segments and a codebook calculated in a training phase. Instead of storing all HOG pattern plus all directional vectors to the feature points as a codebook, we introduce eigen-HOGs (EHOGs) and eigendirectional vectors (EDVs) and propose a completely new training and nonparametric inferring procedure based on a compact codebook containing these EHOGs and EDVs, which allows significantly reducing the memory requirement for the codebook and enables running in hardware of low performance, for example, mobile applications. Our new inferring procedure computes for every image segment a set of directional vectors pointing the prospective feature point positions. Using the compact codebook, such directional vectors are not simply the best match but a linear combination of EDVs where the coefficients for these combinations are derived from the projection of the HOG to the EHOG space. Such a computation does not rely on a computationally expensive search algorithm and therefore is very efficient. We show in our experiments that linearly combining EDVs is a better choice, in the sense of accuracy, than simple taking the best match. As we will show in the experiments, our new inferring procedure is able to handle occlusions efficiently and robustly.

*Related Work*. Recently, several methods [4–12] for finding locations of facial feature points, for example, corners of eyes, eye brushes, mouth, nose tip, beyond detection, and tracking of region center and area have been proposed because of the needs for more accurate recognition of user's subtle intension like expression. They can be categorized into two different methodologies, that is, iterative parameter optimization and nonparametric localization.

Iterative parameter optimization methods [5–9] define the facial feature localization as a parameter optimization problem and solve it by iteratively finding least squares solution for a predefined error metric. Active shape model (ASM) [5, 6], active appearance model (AAM) [7–9], and 3D morphable model (3DMM) [10] are the statistical models which are widely used for facial feature localization by finding optimal parameters of shape and 2D or 3D texture. Although they show good results with some constraints on parameters, they need to set an initial position near optimum and iterative minimization of energy function. In particular, calculation of larger sized hessian matrix is requisite for fitting 3DMM and this causes high computational complexity.

As a nonparametric localization method, Chen et al. [11] calculated the positions of facial feature points by using pixel likelihood maps. They divided facial region into several segments corresponding to each feature point. Then, likelihood maps for each feature point related to the image segments were calculated by using the classifier trained by boosting strategy. This algorithm showed very accurate localization result and fast performance with illumination and scale invariance. However, their method needs a large bundle of nonface training images and cannot deal with occlusion. Kozakaya et al. [4] proposed the codebook-based approach which uses relative directional vectors from regularly divided segment of face image to feature points. They made a codebook which consists of histogram of oriented gradient (HOG) [12] patterns of all training segments, HOG patterns of all facial feature points, and relative directional vectors from all segment centers to facial feature points. For the input face image, they divided face images into canonical segments and found the most similar one from codebook by comparing HOG patterns of input and training segments with approximated nearest neighbor search (ANNS) algorithm [13]. Finally, they calculated the positions of facial feature points by weighted vector concentration (WVC) algorithm which calculates the crossing points of the relative direction vectors corresponding to the found segments. Their algorithm showed a better performance in accuracy than the extended ASM (STASM) [6], the state-of-the-art. Additionally, it could find the occluded feature points from the nonoccluded segments. However, a huge size of storage for codebook, about 600 megabytes for thousands of training images, is inevitable because codebook size is proportional to the number of training segments while a large number of training segments are necessary for generalized performance.

**Figure 1:**
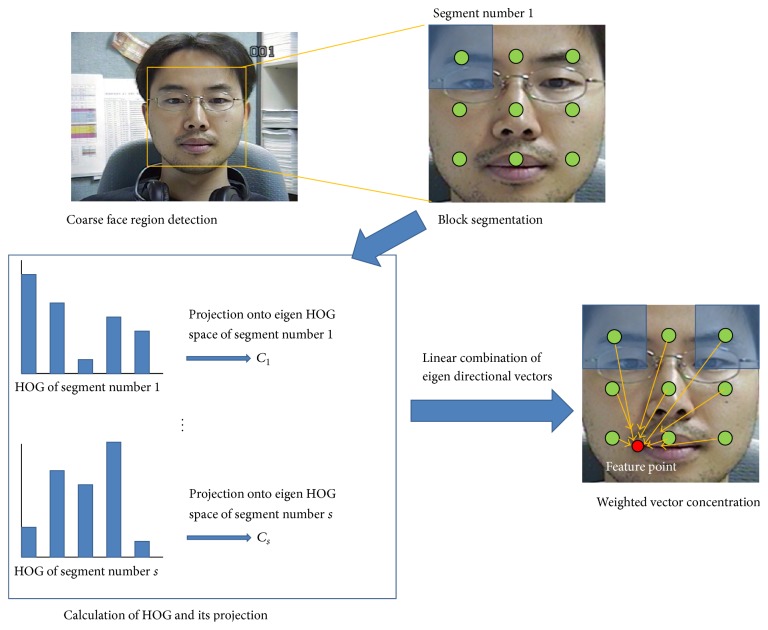
The proposed facial feature localization method. Yellow rectangle on the top left image represents coarse face region from Viola-Jones face detector. The green spots near eyebrows are centers of segments. The red spot on the bottom right image is a feature point, left corner of lip. The orange arrows are the directional vectors from segment centers to feature point.

*Overview*.  Figure 1 shows the proposed facial feature localization frame work. First, coarse face region on the input face image is detected by Viola-Jones face detector [1]. And then the image inside the detected region is resized into a canonical size and segmented into a number of regularly positioned blocks. HOG of each block is calculated and projected onto the EHOGs space of that segment to get the projection coefficients. With these coefficients, directional vector from segment center to feature point is calculated by linear combination of EDVs. Here, whether the block is occluded or not is concluded by checking these projection coefficients and similarity measure of the trained and the input HOG patterns. Finally, the position of feature point is calculated by WVC [4].

The remainder of this paper is organized as follows.  Section 2 shortly repeats how to compute histograms of oriented gradients (HOG). Performing a principal component analysis (PCA) on the HOGs, we compute EHOGs and EDVs in order to train the compact codebook (Section 3).  Section 4 describes the localization procedure of facial features given a single input image. Experimental results are presented in Section 5 and we conclude this work in Section 6.

## 2. Histograms of Oriented Gradients

HOG is a well-known feature for its illumination invariance and high distinctiveness [12]. Similar to [4], we detect the facial region in each training and input image using the Viola-Jones face detector [1]. We resize the detected facial region to 72 × 72 pixels and place the centers of partial overlapping image segments on a regular 9 × 9 grid, which defines a set of 81 image segments. Here, resizing is for no waste of pixel on the face image in calculating HOG and the resizing does not affect the image ratio. For each image segment *s*, we compute a histogram of oriented gradients HOGs. As in [4, 12, 14] experimentally validated, we use three unsigned orientation bins and 3 × 3 blocks of 4 × 4 cells of 5 × 5 pixels, such that each segment has 30 × 30 pixel.  Figure 5 shows the canonical centers of the segments and the cell division. We extract the histogram of each cell by adding the magnitude of each of the pixels gradients to the bin corresponding to its orientation. The histograms of all cells within one block are concatenated and the resulting histogram is normalized. The final histogram is computed by concatenating the histograms of all blocks which is also normalized. See [12] for more details.

## 3. Compact Codebook: Computing Eigen-HOGs (EHOGs) and Eigendirectional Vectors (EDVs)

Using HOG patterns themselves requires a huge amount of memory because it needs to store all the high dimensional patterns, that is, HOG patterns of training segments, HOG patterns of feature points, and their corresponding directional vectors. Several hundred megabytes of storage space for thousands of training images is inevitable for that. Since such extensive memory consumption is far too much for a facial feature localization application, we propose to use principal component analysis (PCA) as a data regression technique to reduce the codebook size.

In order to compute the compact codebook, we collect a set of histograms of oriented gradients from *n* training images. For training image *i* and segment *s*, we extract an HOG *h*_*i*_^*s*^. We assume that facial features have been assigned such that we can store for image *i* a set of directional vectors *v*_*i*_^*s*,*k*^ which points from the center of segment *s* to the feature point *k*. Here *k* is the index of one of the *K* manually annotated feature points. From the histogram data, we construct for each segment *s* a matrix *M*_HOG_^*s*^ = [*h*_1_^*s*^,…, *h*_*n*_^*s*^] where the columns contain the histograms *h*_*i*_^*s*^ obtained from the n training images. Running PCA on such a matrix extracts an average histogram *h*_avg_^*s*^ and eigenhistograms *h*_*e*1_^*s*^ ⋯ *h*_*eD*_^*s*^, with *D* = *n* − 1. We assume that the eigenhistograms are sorted according to their significance; that is, *λ*_*e*1_^*s*^ > ⋯ > *λ*_*eD*_^*s*^, where *λ*_ei_^*s*^ is the eigenvalue of the eigenhistogram *h*_ei_^*s*^. To reduce dimensionality, only the *P* most significant eigenvectors are stored in the columns of a matrix *M*_EHOG_^*s*^ = [*h*_ave_^*s*^, *h*_*e*1_^*s*^,…, *h*_*eP*^*s*^_^*s*^]. Here *P* ^*s*^ is chosen such that at least a fixed fraction *f* (e.g., 0.9) of variance (energy) is preserved:

(1) $$\begin{array}{c} f\leq \frac{\sum_{d=1}^{P^{s}}\lambda_{ed}^{s}}{\sum_{d=1}^{D}\lambda_{ed}^{s}}. \end{array}$$

After PCA, EHOGs can be represented as a linear combination of HOGs

(2) $$\begin{array}{c} M_{HOG}^{s}\cdot C^{s}=M_{EHOG}^{s}, \end{array}$$

where *C* ^*s*^ ∈ *ℜ* ^*n*×(*P*^*s*^+1)^ is a coefficient matrix which is computed as

(3) $$\begin{array}{c} C^{s}={\left(\;{M_{HOG}^{s}}^{T}\cdot M_{HOG}^{s}\;\right)}^{-1}{M_{HOG}^{s}}^{T}\cdot M_{EHOG}^{s}. \end{array}$$

**Figure 2:**
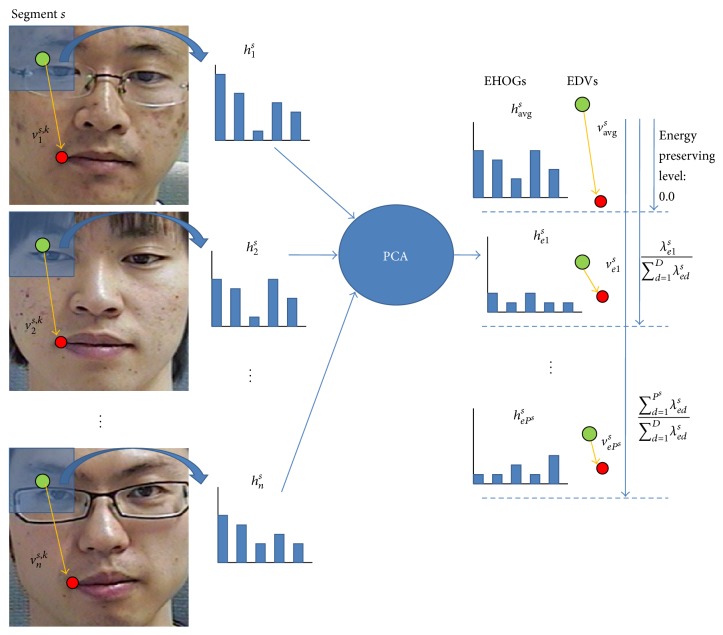
Calculating EHOGs and EDVs.

We use this coefficients matrix to compute the eigendirectional vectors for every facial feature point *k* and segment *s* as

(4) $$\begin{array}{c} V_{EDV}^{s,k}=V^{s,k}\cdot C^{s}, \end{array}$$

where *V* ^*s*,*k*^ = [*v*_1_^*s*,*k*^,…, *v*_*n*_^*s*,*k*^] ∈ *ℜ* ^2×*n*^ and *V*_EDV_^*s*,*k*^ = [*v*_ave_^*s*,*k*^, *v*_*e*1_^*s*,*k*^,…, *v*_*eP*^*s*^_^*s*,*k*^] ∈ *ℜ* ^2×(*P*^*s*^+1)^. The whole procedure of codebook making is depicted in Figure 2.

## 4. Localizing Facial Features

In what follows, we describe how we compute the position of facial features in a new input image, as during the training we detect the facial region using the Viola-Jones face detector [1]. As pointed out in Section 2, we resize this region to 72 × 72 pixels and regularly position the centers of 9 × 9 segments in the resampled face region. Then, we compute histograms of oriented gradients *h* ^*s*^ for each segment *s*. Each HOG can be approximated as a linear combination of EHOGs where the coefficients are obtained by a simple and fast projection step. We use these coefficients to compute directional vectors to all facial features as a linear combination of EDVs and use a similarity-based and a distance-based integration approach to infer the positions of the facial features. As the final step, the localization result in 72 × 72 pixel sized image is resized into the original image size.

### 4.1. Computing Directional Vectors from Face Segments to the Feature Points

In our method, we do not rely on any time consuming search algorithm like ANNS [13] to compute the best matching HOG from the codebook. In what follows we describe how we compute the *K* directional vectors to the feature points originating from the segment *s*. Assuming the HOG of this segment to be *h* ^*s*^, we can compute coefficients [*c*_1_,…, *c*_*P*^*s*^_] such that

(5) $$\begin{array}{c} h^{s}\approx h_{ave}^{s}+\overset{P^{s}}{\underset{i=1}{\sum }}c_{i}^{s}h_{ei}^{s}. \end{array}$$

The coefficients can be computed by projection as *c*_*i*_^*s*^ = 〈*h* ^*s*^ − *h*_ave_^*s*^, *h*_ei_^*s*^〉, where 〈*a*, *b*〉 represents the inner product.

In order to compute the directional vector *v* ^*s*,*k*^ from the center of the segment to the feature *k*, we use these coefficients to linearly combine the EDVs:

(6) $$\begin{array}{c} v^{s,k}=v_{ave}^{s,k}+\overset{P^{s}}{\underset{i=1}{\sum }}c_{i}^{s}v_{ei}^{s,k}. \end{array}$$

**Figure 3:**
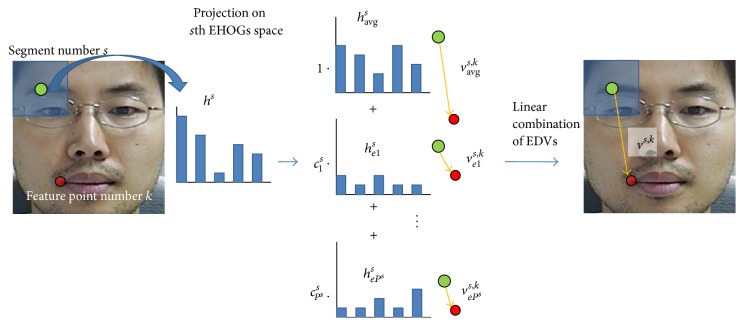
Calculating the directional vector of a segment.

Figure 3 shows the procedure of calculating a directional vector.

### 4.2. Computing Final Feature Point Positions by a Weighted Vector Concentration (WVC)

After computing the directional vectors pointing from the segments centers to the feature points, WVC [4] is used to calculate the positions of feature points such that they have the least weighted sum of squared distances from the lines of directional vectors as

(7) $$\begin{array}{c} \underset{x_{k},y_{k}}{\arg\;\min}\overset{M}{\underset{s=1}{\sum }}w_{s}^{2}{\left\|\;a_{s}x_{k}+b_{s}y_{k}+c_{s}\;\right\|}^{2}, \end{array}$$

where *w*_*s*_ is the weight of segment *s*, (*x*_*k*_, *y*_*k*_) is the coordinate of facial feature point *k*, (*a*_*s*_, *b*_*s*_, *c*_*s*_) is the coefficients of linear equation *a*_*s*_ *x* + *b*_*s*_ *y* + *c*_*s*_ = 0 with *a*_*s*_^2^ + *b*_*s*_^2^ = 1 representing the line going through the center of segment *s* and feature *k*, and *M* is the total number of facial segments.

For the weights of segments, first we define the similarity-based weight for the directional vector of segment *s* as the inner product of *h* ^*s*^ and its projection onto the *s*th EHOGs space:

(8) $$\begin{array}{c} w_{s}^{}=\langle \;h^{s},h_{ave}^{s}+\overset{P^{s}}{\underset{i=1}{\sum }}c_{i}^{s}h_{ei}^{s}\;\rangle . \end{array}$$

The dot product of the HOG extracted at the prospective feature position with the HOG at the feature position observed in the training data is a good indicator for the correctness of the directional vector if the face is not occluded [4]. However, if the input face image is partially occluded, the HOG extracted at the occluded prospective feature position is much different from the trained one even though the HOG patterns of the corresponding image segments are very similar to each other. Note that we do not extract an HOG at positions predicted by each directional vector to compute *w*_*s*_, which is computationally quite expensive since we need to extract *K* × *S* of HOGs. We rather use our quality measurement based on the Mahalanobis distance which measures the similarity of unknown samples to known ones. Compared to the eigenvalues *λ*_*e*1_^*s*^, …, *λ*_*eP*^*s*^_^*s*^, if one of the projection coefficient squares *c*_*ej*_^*s*^ exceeds 2.5-sigma (standard deviation) bound, we infer that the observed HOG is far away from the distribution of HOG of the corresponding segment. In such case, we explicitly set its weight to zero

(9) $$\begin{array}{c} w_{s}^{}=\left\{\begin{array}{cc} w_{s}^{s} & if\;\;\left|c_{ej}^{s}\right|<2.5\sqrt{\lambda_{ej}^{s}}\;\;for\;\;j=1\cdots P^{s},\\ 0 & otherwise. \end{array}\right. \end{array}$$

The second weight is based on distance measure in position. We use Gaussian kernel function among the kernel functions of square distance to give larger weights to the segment closer to the feature point than those far from the feature point

(10) $$\begin{array}{c} w_{s}^{}=w_{s}^{s}\cdot e^{-d^{2}/L^{2}}, \end{array}$$

where *d* is the distance between segment center and feature point and *L* is the half length of rectangular region of face.

The remainder of the WVC procedure goes through LMedS [15] and distance-based weighting which are same to the WVC procedure of [4].

## 5. Experimental Results

In this section, we compare our method using EHOGs and EDVs to the original codebook approach [4] which used HOGs and a nearest neighbor search in storage size, localization accuracy, and processing speed. For all experiments, we used a Pentium 4 PC with a 2.6 GHz Quad core CPU and 2 GB memory.

### 5.1. Training

**Figure 4:**
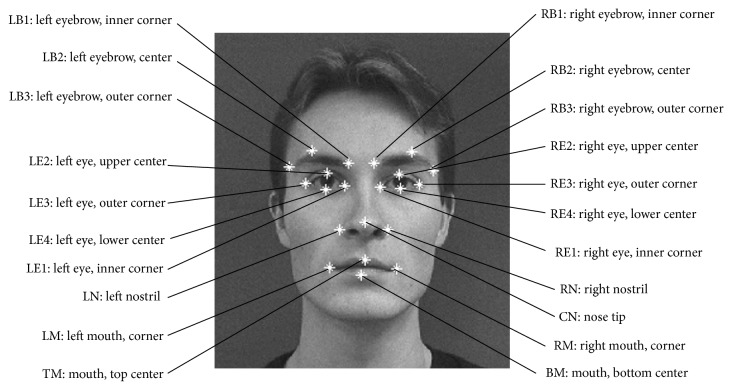
Definition of 21 facial feature points.

We gathered 969 upright frontal-view face images from various sources by using Viola-Jones face detector [1]. We manually marked 21 facial feature points like eyes, eyebrows, nose, and mouth (see Figure 4). These images and facial feature points were used as the training data to make EHOGs and EDVs.

**Figure 5:**
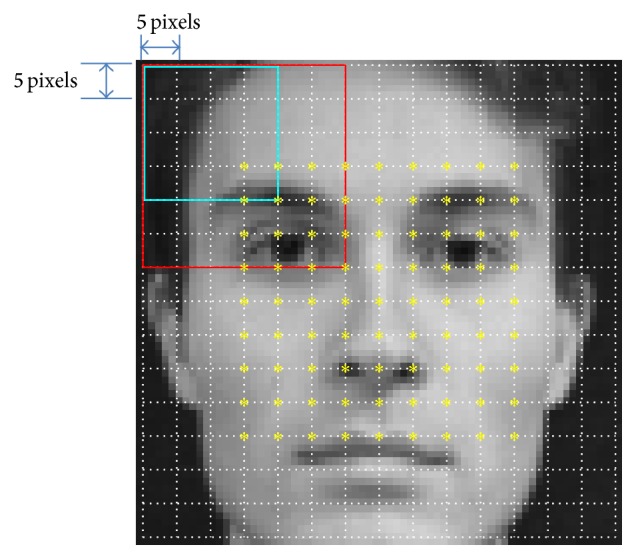
Canonical segmentation of face image. Yellow asterisks stand for the centers of segments, white stitched rectangles stand for cells, cyan rectangle stands for block, and red rectangle stands for segment.

For an HOG descriptor of a segment, we used three unsigned orientation bins and 3 × 3 blocks of 4 × 4 cells of 5 × 5 pixels which was determined by preliminary experiment in [4, 14]. Therefore, each segment has 30 × 30 pixel size and each face image has 9 × 9 segment array.  Figure 5 shows the canonical centers of segments and cell division. The first and the last rows and columns of pixel are not used because HOG cannot be calculated on them due to the lack of gradient. 78489 (969 images and 81 segments per image) of local HOG patterns and 1648269 (969 images, 81 segments per image, and 21 feature points per segment) of directional vectors for all the training segments were calculated with this parameter setting. Additionally, 20349 (969 images and 21 feature points per image) of local likelihood HOG patterns [4] for all facial feature points were calculated. The set of the local HOG patterns, the directional vectors, and the local likelihood HOG patterns was stored as an original codebook [4] for the purpose of performance comparison. Next, the local HOG patterns and the directional vectors went through a further process which is explained in Section 3 to make EHOGs and EDVs for each segment with varying energy preserving level.

### 5.2. Storage Size

**Figure 6:**
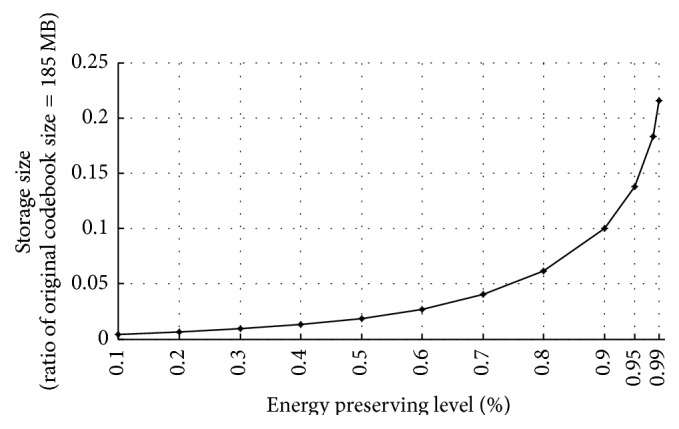
Storage size versus energy preserving level.

Figure 6 shows the required storage size for EHOGs and EDVs versus energy preserving level of PCA. While the original codebook needs about 185 MB of storage size, the storage size of our method is drastically reduced as the energy preserving level decreases. When the energy preserving level is 70%, the storage size reaches 20 times less size than original codebook. Even when the energy preserving level is 99%, the storage size is less than a quarter of original codebook size. This means that the HOG pattern space of facial image segment has a large amount of redundancy and that applying PCA effectively saves the storage size.

### 5.3. Test of Feature Point Localization without Occlusion

We evaluated the proposed method (EHOGs + EDVs) with FERET duplicate I dataset [16] and compared its performance with that of HOG + NNS [4]. The probe set of FERET which consists of 722 images was selected for test and we manually marked 21 facial feature points on the images as the ground truth. Note that the training set in Section 5.1 is independent of this test set. As the first step of localization test, detected face regions on FERET face images by using Viola-Jones face detector [1] were resized into 72 × 72 pixels. Then, with the same face region information, our method and HOG + NNS method were applied to detect 21 facial feature points. Here, we used approximation error *ε* = 0 for ANNS for the best matching result with exact NNS.

**Figure 7:**
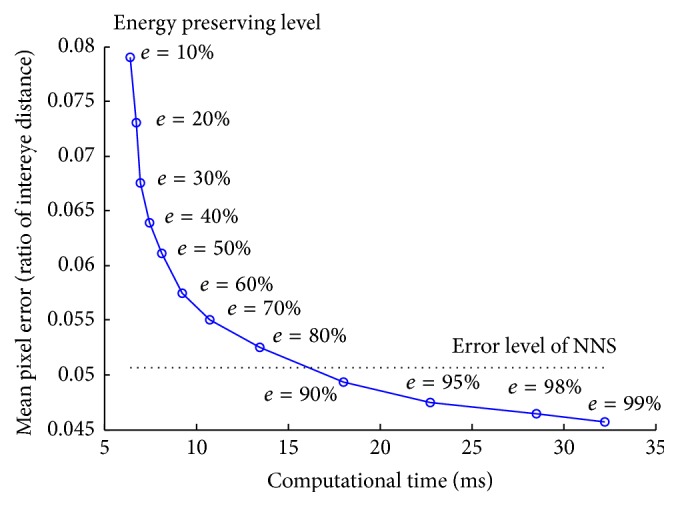
Localization error versus computation time.

Figure 7 shows how the feature localization error and the computation time change according to the energy preserving level of the proposed method. The computation time represents the average time consumption for HOG calculation and directional vector calculation on an input image. Detection error was measured as the pixel distance between the detected feature points and the manually marked ground truth. The mean pixel error was calculated as the average of the pixel distances divided by the distance between two eye centers. As the energy preserving level goes down from 90% to 10%, the computation time also goes down because the less number of EHOGs and EDVs requires the less computational cost for directional vector calculation in Section 4.1. “Error level of NNS” (0.0507) is the localization error obtained by using original codebook of HOG pattern and NNS, which is the best performance with original codebook. Compared to this error level, energy preserving level more than 80% is allowable because it maintains the accuracy above 97% of “error level of NNS.”

**Figure 8:**
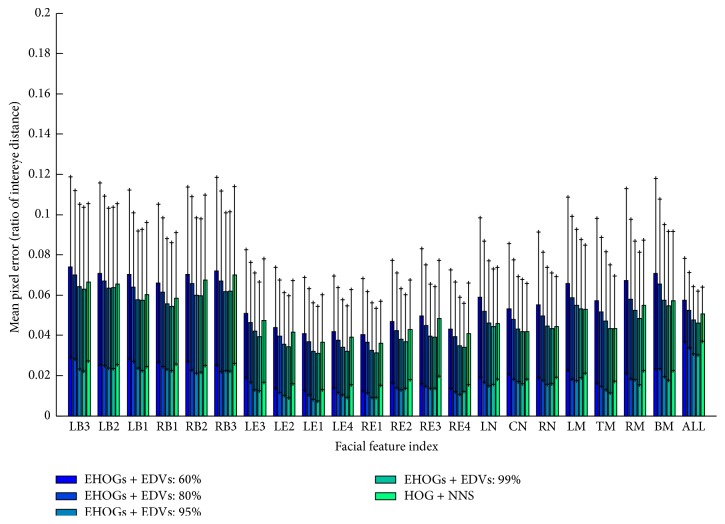
Localization errors and standard deviations of facial feature points without occlusion. The label of feature index is same to the definition on Figure 4. The bars labelled as “ALL” represent the average errors and standard deviations for the tested methods.

Note that the localization error of the energy preserving level above 90% is smaller than the “error level of NNS.” This is very impressive result because the proposed method not only reduces storage size but also improves localization accuracy. This seems to be happening because the proposed algorithm calculates more accurate directional vector by integrating the directional vectors of the training segments while nearest search algorithm finds the best matched pattern in codebook and just uses the directional vector of that matched pattern. Therefore, the proposed method with high energy preserving level performed better by utilizing the statistical characteristic of segments than the original codebook-based approach [4] did.  Figure 8 shows the localization result for each facial feature point. Average errors (and standard deviations) for the proposed method (EHOGs + EDVs) with 60%, 80%, 95%, and 99% and the original codebook-based method with exact nearest neighbour search algorithm (HOG + NNS) are 0.0572 (0.0193), 0.0526 (0.0189), 0.0475 (0.0167), 0.0457 (0.0156), and 0.0507 (0.136).

### 5.4. Test for Facial Feature Localization with Partial Occlusion

For the test of robustness against partial occlusion, we put a white block on the test images of Section 5.2. The size of the white block was 10% of the area of input facial region and its positions were randomly set on the facial region. Then, we applied our method and the HOG + NNS to these occluded images.

**Figure 9:**
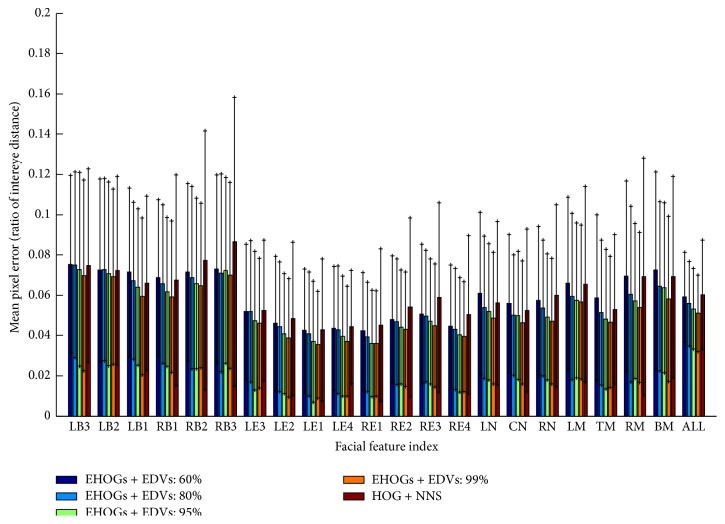
Localization errors and standard deviations of facial feature points with 10% occlusion. The label of feature index is same to the definition on Figure 4. The bars labelled as “ALL” represent the average errors and standard deviations for the tested methods.

The result of the facial feature localization with occlusion is shown in Figure 9. The tendency of average accuracy is similar to the nonoccluded case (Figure 8). However, the increment of error is higher with HOG + NNS than EHOGs + EDVs. In particular, for the right eye brow (RB3), right eye (RE1-4), and mouth (LM, RM, and BM), the error increment is much higher than the proposed method. As explained in Section 4.2, HOG + NNS used similarity measure of local likelihood pattern of facial feature point which is occluded and resulted in higher error increment than our proposed method which used HOG pattern of segment to give the nonoccluded segment a high certainty.

**Figure 10:**
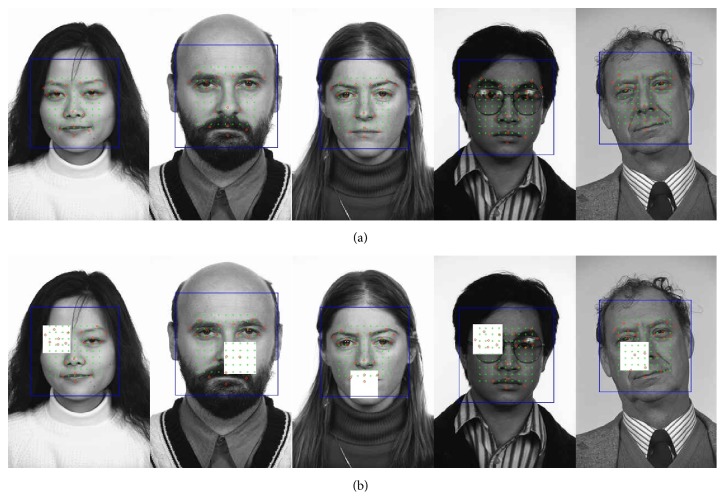
Sample results of the proposed facial feature localization with 90% energy preserving level on FERET face images without (a) or with 10% occlusion (b).

Figure 10 shows several sample results of the proposed facial feature localization with 90% energy preserving level without or with 10% occlusion. The images on top row are the results without occlusion and those on bottom row with occlusion. The blue rectangle represents coarse face region detected by Viola-Jones face detector [1], green dots represent the segment centers, and red dots represent the detected facial feature points. The proposed method localized the feature points correctly on nonoccluded faces. And even on occluded faces, it estimated the locations of the occluded feature points from directional information of the nonoccluded segments. For the fifth column of images of Figure 10, the face is rotated by 20 degrees from upright position and this caused incorrect localization of feature points because our training database does not have any rotated images. This can be easily dealt with some existing techniques of image alignment or learning with augmented training data. More about this will be discussed in conclusion session.

### 5.5. Overall Performance Comparison

The time consumption, the required storage size, and the accuracy of the tested algorithms are summarized in Table 1 for the performance comparison at a glance. Our method is very fast, accurate, and low storage consuming over HOG + ANNS (*ε* = 10 as [4]) and HOG + NNS. Because they need much more calculations of HOG patterns of the prospective facial feature points (81 segments × 21 feature points = 1701 calculations) than our method, they need hundreds of milliseconds in HOG calculation. EHOGs + EDVs with 80% energy as the best choice of our method with regard to the balance between storage size and accuracy has 16 times less storage and a little smaller error than HOG + ANNS method.

## 6. Conclusion

In this work, we proposed a new algorithm to localize facial feature points. Adopting the framework of original codebook method [4], we introduced a new way to deduce directional vector pointing to facial feature without using any time consuming search algorithm. First we employed PCA to learn a compact codebook from a set of HOGs extracted at regular placed segment within the facial region and directional vectors pointing from these segments to manually selected facial feature points. Second, a simple linear combination of segmental directional vectors allowed computing the directions to the feature points. Third, the energy preservation level was used to control the tradeoff between storage size and accuracy of the localization. By comparing our method to the original codebook method, we experimentally justified that our method with an energy preservation level around 80% drastically reduces the memory consumption (94% of the original codebook is saved) and time consumption while producing a comparable localization error. Our new occlusion handling is based on the distribution of the HOGs in the training data and we also experimentally showed that this approach handles occlusions very robustly.

We start the facial feature localization from the detected coarse face region which is aligned to upright. Since our database does not contain pose varying faces like one shown in the right-most column of Figure 10, we have problems to infer the correct feature positions in such cases. One could easily tackle the in-plane rotation problem by adopting image registration techniques [17, 18] as the prior step of our compact codebook approach. In particular, because generalized hyperplane approximation (GHA) [18] achieved very good alignment result by regression learning and HOG is also used in this approach, using GHA is better in computation time and algorithm consistency. For the out-of-plane rotation problem, we can add pose variations of somewhat profile or out-of-plane rotated faces to the training database. This has been done by the machine-learning-based method like regression learning [19] or neural network approach [20] and achieved competitive out-of-plane rotation invariant results. But adding more samples to training data will make additional bases in eigen-HOGs or eigendirectional vectors and this will give some increase in codebook storage. We focused on compact codebook and eigenfeatures in this work and the combination with those methods and more analysis about this remained as a future work.

## Figures and Tables

**Table 1:** Performance comparison of facial feature localization.

Method∖Process	HOG calculation	Directional vector EDVs or ANNS or NNS	WVC	Total time consumption	Storage size (%)	Average error/standard deviation (with 10% occlusion)
EHOGs + EDVs 60%	3 ms	3 ms	5 ms	11 ms	5.0 MB (2.7%)	0.0572/0.0193 (0.0593/0.0213)
EHOGs + EDVs 80%		7 ms		15 ms	11.4 MB (6.2%)	0.0526/0.0189 (0.0571/0.0204),
EHOGs + EDVs 95%		16 ms		24 ms	25.5 MB (13.8%)	0.0475/0.0167 (0.0515/0.0192),
EHOGs + EDVs 99%		25 ms		33 ms	39.8 MB (21.5%)	0.0457/0.0156 (0.0506/0.0189),
HOG + ANNS [4] (*ε* = 10) HOG + NNS (ANNS with *ε* = 0)	483 ms	16 ms 1490 ms		499 ms 1978 ms	185.3 MB (100%) 185.3 MB (100%)	0.0527/0.0151 (0.0631/0.0185) 0.0507/0.0136 (0.0602/0.0174)
